# Cellular morphological changes detected by laser scanning *in vivo* confocal microscopy associated with clinical outcome in fungal keratitis

**DOI:** 10.1038/s41598-019-44833-9

**Published:** 2019-06-06

**Authors:** Jaya D. Chidambaram, Namperumalsamy V. Prajna, Srikanthi Palepu, Shruti Lanjewar, Manisha Shah, Shanmugam Elakkiya, Prajna Lalitha, David Macleod, Matthew J. Burton

**Affiliations:** 1 London School of Hygiene & Tropical Medicine, Keppel Street, London, WC1E 7HT UK; 2 Aravind Eye Hospital, 1 Anna Nagar, Madurai, 625 020 Tamil Nadu India; 3 Aravind Medical Research Foundation, Kuruvikaran Salai, Anna Nagar, Shenoy Nagar, Madurai, 625020 Tamil Nadu India; 4 Cornea Department, Moorfields Eye Hospital, 162 City Road, London, EC1V 2PD UK

**Keywords:** Fungal host response, Prognostic markers

## Abstract

HRT3 *in vivo* confocal microscopy (IVCM) images may indicate clinical outcome, but few studies have analysed this in fungal keratitis (FK). Adults with FK (diameter ≥3 mm) presenting to Aravind Eye Hospital, India from 2012-3 were enrolled prospectively. IVCM was performed at baseline, days 7, 14 and 21 post-enrolment (+/− 3 days where possible). Specific morphologies were identified in IVCM images by a grader masked to microbiology and clinical outcome (defined as good: healed/improving, or poor: enlarged ulcer, perforation or transplant/glue). Associations with final visit outcome assessed using logistic regression. 143 FK participants were enrolled; 87 had good outcome, 56 had poor outcome. Poor outcomes were associated with stellate interconnected cellular processes with no visible nuclei (OR 2.28, 95% CI: 1.03–5.06, p = 0.043) in baseline IVCM images, and fungal filaments (OR 6.48, 95% CI:2.50–16.78, p < 0.001) or honeycomb distribution of inflammatory cells (OR 5.24, 95% CI: 1.44–19.06, p = 0.012) in final visit images. Fungal filaments (OR 3.61, 95% CI:1.64–7.95, p = 0.001), stromal dendritiform cells (OR 2.88, 95% CI:1.17–7.11, p = 0.022), or stellate cellular processes with no visible nuclei (OR 2.09, 95% CI:1.14–3.82, p = 0.017) were associated with poor outcome if not in baseline but present in final visit images. IVCM can reveal morphological changes associated with clinical outcome.

## Introduction

After initiation of antimicrobial therapy for microbial keratitis (MK), it may be difficult to assess therapeutic response of some ulcers based upon clinical appearances alone^1^. *In vivo* confocal microscopy (IVCM) is a useful aid in detecting pathogens >1μm in size in the living cornea, i.e. fungal hyphae and *Acanthamoeba* cysts but not most bacteria^2^. Specific cellular morphologies observed with IVCM, such as increase in the number of activated keratocytes have been previously postulated as early predictors of disease, e.g. graft rejection after penetrating keratoplasty^3^. Only a few studies have used IVCM to assess changes during the clinical course of MK^1^. Winchester *et al*. used tandem-scanning IVCM in two patients with fungal keratitis (FK) and showed that *Aspergillus* hyphae broke into shorter fragments by day 4 post-natamycin therapy, and disappeared completely by day 12^4^. Shi *et al*. used the confoscan IVCM to monitor 121 FK patients during antifungal therapy, and observed that fungal hyphae disappeared over time in parallel with reduction in the inflammatory cell infiltrate, however no quantitative data were presented on the association of specific IVCM cellular changes with clinical outcome^5^. Heidelberg retinal tomography 3 IVCM with the Rostock Corneal Module (HRT3 IVCM) has a higher resolution than either the tandem-scanning or confoscan IVCM and therefore allows an improved view of cells within corneal tissue^6^. Cellular processes and nuclei of activated keratocytes can be observed in greater detail using the HRT3 IVCM^6^, as well as other morphological appearances such as presence of round inflammatory cells and dendritiform cells.

To our knowledge, use of serial HRT3 IVCM to assess the cellular changes in MK has not been previously reported. Therefore, in this study we quantitatively assessed serial HRT3 IVCM images from a prospective cohort of severe bacterial and fungal keratitis patients in South India for morphological features that were associated with healing or worsening clinical outcome. We chose to focus on moderate-to-severe keratitis in this study since these ulcers often present with an atypical clinical appearance and may be difficult to manage during their clinical course^7^, therefore we felt these ulcers would benefit the most from investigation of the cellular response with the use of IVCM.

## Methods

This study was prospectively approved by the Ethics Committees of the Indian Council for Medical Research, Aravind Eye Hospital, Tamil Nadu, India, and the London School of Hygiene & Tropical Medicine, UK. All experiments were carried out in accordance with the relevant guidelines and regulations. All patients gave written informed consent prior to enrolment; illiterate participants gave informed consent with a witnessed thumbprint on the study consent form.

From February 2012 to February 2013, consecutive patients presenting to the Cornea Clinic of Aravind Eye Hospital, Madurai, Tamil Nadu, India were assessed for eligibility with the following inclusion criteria: age ≥18 years, stromal infiltrate diameter ≥3 mm, presence of overlying epithelial defect and signs of acute inflammation. Patients were excluded if the ulcer had a descemetocoele or >80% corneal thinning as assessed by slit lamp examination (since applanation for IVCM could not safely be done in these patients); clinical features or prior history of herpetic keratitis; Snellen visual acuity worse than 6/60 in the unaffected eye; *Acanthamoeba* keratitis diagnosed on culture and/or IVCM (these patients were followed up in a different study); or presence of mixed infection (i.e. culture-positive for bacteria and light microscopy and/or IVCM positive for fungus). At enrolment, data from a focused clinical history and slit lamp examination were recorded. The study follow-up visit schedule was as follows: days 7 (+/− 3 days), 14 (+/− 3 days) and 21 (+/− 3 days). Final visit assessment with IVCM imaging was included for patients unable to attend at 21 (+/− 3 days), but who attended later up to a maximum of 37 days post-enrolment. Additional assessments were scheduled as clinically indicated. At each visit, the cornea consultant or cornea fellow examined every study participant and management followed the standard of care for microbial keratitis at Aravind Eye Hospital (AEH). The standard of care for the treatment of fungal keratitis at AEH is as per the World Health Organization’s guidelines for the management of microbial keratitis^8^, i.e. intensive topical natamycin 5% eyedrop therapy every 1 hour day and night (with hospital admission for severe ulcers), and drop frequency slowly tapered as per clinical response. In addition to topical therapy, oral antifungal medication (e.g. ketoconazole, itraconazole or voriconazole) was added in cases of large, deep ulcers or ulcers close to the limbus. Adjunctive therapy with topical cycloplegic agents or anti-glaucoma medications were given as required. Where there was clinical suspicion of bacterial infection, empirical therapy with intensive topical antibiotic (e.g. fluoroquinolone such as moxifloxacin or levofloxacin, or fortified cefazolin or fortified gentamicin) was used in addition to the antifungal therapy, as per the recommendation of the corneal specialist. The antibiotic was then tapered and/or stopped as per clinical response and along with availability of microbiological results from corneal scrape testing. Any participant with significant worsening of the ulcer despite maximal medical therapy (e.g. perforation or impending perforation, significant enlargement of the infiltrate) underwent surgical intervention (i.e. therapeutic penetrating keratoplasty; corneal glue for small perforations; intrastromal voriconazole for deep stromal abscess in fungal keratitis).

Clinical outcomes were assessed based on slit lamp examination of the cornea including measurements of the ulcer at each visit with grading as follows: (1) healed: no epithelial defect at final visit with presence of scar tissue; (2) improving: final visit infiltrate size less than enrolment size with or without epithelial defect still present; (3) worsening: final visit infiltrate size either the same as or larger than enrolment infiltrate size, if corneal perforation was noted (i.e. full-thickness hole seen in cornea at slit lamp, or iris plugging site of possible corneal perforation with flat anterior chamber and seidel-positivity), or if surgical intervention was performed. The “final visit” was defined as either the clinical assessment/IVCM imaging performed at the end of the study (i.e. up to 37 days post-enrolment) or the visit when the patient left the study due to surgical intervention. Ulcers were categorized as having a “Good” outcome if they healed or were improving, and categorized as having a “Poor” outcome if they had worsened or perforated by the final visit.

### IVCM Imaging

HRT3 IVCM imaging (Heidelberg Engineering, Germany) of the corneal ulcer was performed immediately prior to corneal scraping for microbiological tests, as described in detail elsewhere^2,9^. Proparacaine 0.5% eyedrop anaesthesia was used (Aurocaine, Aurolab, Madurai, India) and the RCM (Heidelberg Engineering, Germany) with 63x objective lens (Nikon, Japan) was gently applanated to the corneal surface. Using the HRT3 IVCM (Heidelberg Engineering, Germany), volume scans were obtained in a systematic manner at the center of the ulcer and ulcer margins (12, 3, 6 and 9 o’clock positions). By manually refocusing the Rostock Corneal Module we obtained a series of volume scans covering consecutive 80 micron depths from epithelium to endothelium, where possible, in each of these cardinal locations at the ulcer margins and center. This imaging method has been previously used to systematically scan the cornea in prior publications^2,10^. IVCM imaging was repeated using the same methodology at each follow-up visit. After IVCM imaging, corneal scrapes were obtained from the leading margin and base of the ulcer for culture and light microscopy, using standard procedures described in detail elsewhere^2^.

### IVCM Grading

**Figure 1:**
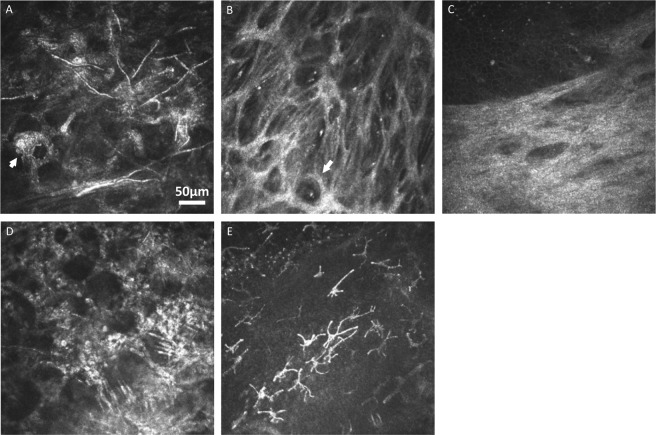
HRT3 *in vivo* confocal microscopy images at final visit showing: (**A**) fungal filaments in the corneal stroma (arrow shows activated keratocytes with granular intracellular contents), (**B**) stromal bullae shown by arrow, and (**C**) acellular scar tissue in lower half of image, (**D**) several “spindles” (indicated by arrow) occurring in parallel, adjacent to each other, and (**E**) dendritiform cells in the anterior stroma.

**Figure 2:**
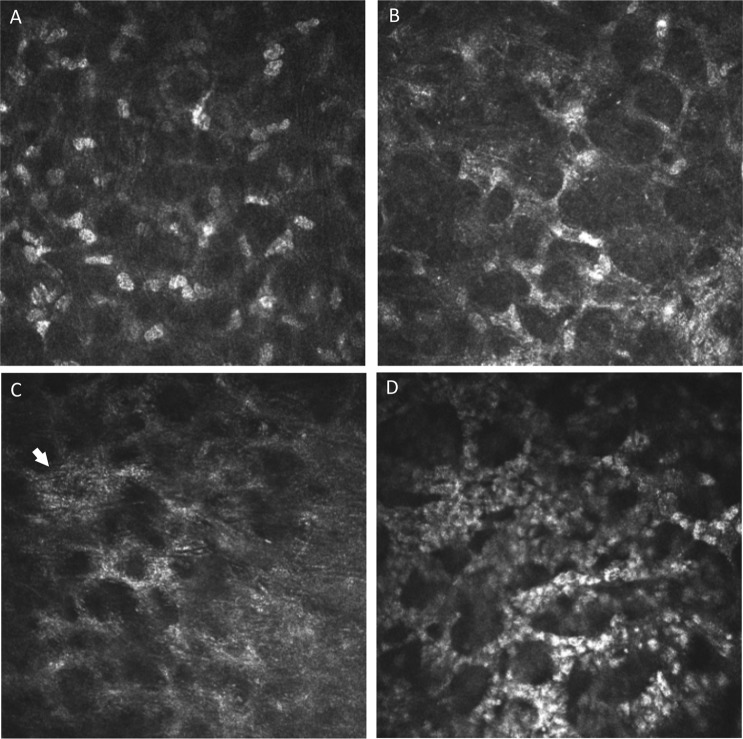
HRT3 *in vivo* confocal microscopy images of keratocytes in fungal keratitis showing: (**A**) normal keratocyte morphology (bright ovoid nuclei with barely visible cellular processes), (**B**) stellate interconnected cellular processes with bright ovoid nuclei (shown by arrow), (**C**) stellate interconnected cellular processes but no visible nuclei (arrow shows granular intracellular appearance), (**D**) inflammatory cells in a honeycomb distribution.

IVCM volume-scan images were assigned a random identification number, had patient-identifying data removed, and were shuffled into a random order. A single experienced grader performed all image grading and was masked to the clinical features and microbiological diagnosis. Images from each visit were graded independently from all other visits. All data were recorded in a Microsoft Access database. The grading scheme included presence/absence of the following morphological features in any single IVCM image as described in Table 1: fungal filaments (Fig. 1A), corneal epithelial or stromal bullae (Fig. 1B), homogenous-acellular scar-like tissue (Fig. 1C), linear “spindles” (Fig. 1D), or dendritiform cells in either the basal epithelial layer or stroma (Fig. 1E). Presence/absence of the following morphological features were also assessed: normal, quiescent keratocytes (i.e. bright ovoid nuclei with barely visible cellular processes; Fig. 2A); stellate structures with bright interconnected broad cellular processes either with or without visible bright ovoid nuclei (“activated” keratocytes, as shown in Fig. 2B,C respectively); inflammatory cells in either a honeycomb distribution (Fig. 2D) or a non-specific distribution; or “granules” (i.e. 1–2μm diameter bright opacities within the nucleus or cellular processes; Fig. 2C).

**Table 1:** Details of the grading scheme used to classify HRT3 *in vivo* confocal microscopy images of fungal keratitis at each visit.

Grading Scheme Component	Morphological Details
Fungi	Linear white hyper-reflective structures, width: 3–6 microns, length up to 400 microns. Often forming a branching pattern (Fig. 1A).
Bullae	Dark hypo-reflective lacunae within epithelial cell layer or stromal layer (Fig. 1B).
Scar tissue	Stromal tissue of homogenous reflectivity. Usually acellular. Occasionally contains bullae (Fig. 1C).
“Spindles”	Very straight linear, hyper-reflective structures with no branching. Often occurring in groups that are parallel to each other (Fig. 1D)
Dendritiform cells	Hyper-reflective cells, with long cellular processes extending outwards in multiple directions from the cell body (Fig. 1E). Usually several present in each IVCM image in moderate-to-severe fungal keratitis cases.
Normal, quiescent keratocytes	Hyper-reflective ovoid nuclei with partially visible cellular processes, connected to cellular processes of adjacent keratocytes in a honeycomb formation (Fig. 2A).
“Activated” keratocytes with visible nuclei	Bright hyper-reflective ovoid nuclei with broad cellular processes that are interconnected forming a stellate appearance (Fig. 2B).
“Activated” keratocytes without any visible nuclei	As above, broad interconnected stellate appearance of keratocyte network of cellular processes but with no visible ovoid nuclei within the cells (Fig. 2C).
“Granules”	Granular appearance of hyper-reflective material (1–2 microns in diameter) within keratocyte cellular processes or within cell nuclei (Fig. 2C).
“Inflammatory” cells	Small, bright hyper-reflective round cells (approx. 10 microns in diameter) often seen in large numbers in the affected tissue. Can form a honeycomb distribution within the corneal stroma (Fig. 2D).

### Statistical methods

All statistical analyses were performed in Stata 13.1 (StataCorp, Texas, USA). Logistic regression analysis was used to assess statistical significance of differences in morphological features in ulcers with poor outcome versus good outcome in the baseline visit IVCM images and then in the final visit images. Conditional logistic regression analysis was used to assess change in morphological features within each patient’s images from baseline to the final visit, with poor versus good outcome as the dependent variable (i.e. although patients may have been followed up at different times for their final visit, this factor is taken into account as the conditional logistic regression analysis only analyses the change within each patient alone, not a comparison between patient groups). All regression analyses were adjusted for age, gender, baseline ulcer stromal infiltrate size (defined as the geometric mean of longest stromal infiltrate diameter and its perpendicular diameter) and time from symptom onset to clinic visit (for analyses that included the final visit data). Forwards stepwise selection was used to create the multivariable model (variables included if p < 0.1 in univariable regression analyses and retained if p < 0.05 in likelihood ratio test with full model with all selected variables included).

## Results

### Participants and microbiology

One-hundred and eighty-two participants with FK were initially enrolled into the study (FK diagnosed as follows: 143 culture-positive, 30 culture-negative/light microscopy positive, and 9 microbiologically-negative/IVCM-positive for fungi.) However, 39 patients were excluded from the study due to the following reasons: 7 lost to follow-up after the first visit; inability to perform IVCM at the final visit in 32 (due to prior corneal perforation in 21, excessive corneal thinning/descemetocele in 3, recent penetrating keratoplasty in 7, and 1 patient unable to attend for IVCM imaging). In the final 143 participants included in the study, a total of 1647 volume scans were performed (median 11 volume scans per patient, interquartile range 8–13), consisting of a total of 65,880 images that were assessed in this study.

Of the patients who reported use of any antimicrobial therapy prior to presentation (n = 126), 23.8% had been using natamycin alone (n = 30/126), 5.5% had been using an azole alone (e.g. voriconazole; n = 7/126) and 27.8% had been using both natamycin and a topical azole in combination (n = 35/126). After being seen in the cornea clinic, the 143 final study participants were commenced on the following intensive (hourly) antifungal eyedrops: 5% natamycin alone in 93, 5% natamycin and 1% voriconazole in 46, topical 1% itraconazole eyedrops in one, initial antibiotic (moxifloxacin) in one until fungal culture results noted at first follow-up visit then natamycin started; fortified cefazolin, levofloxacin and polymyxin B ointment in one patient whose ulcer had resolved by the time fungal results were available (i.e. culture-negative, equivocal IVCM images at presentation, but found to have few fungal filaments on IVCM imaging at a later visits once clinically resolving). An oral antifungal agent was given in 103 patients (e.g. itraconazole, ketoconazole, or fluconazole tablet) during the study. In ulcers that were found to be clinically worse or not improving at follow-up visits, antifungal treatment was changed as follows: topical voriconazole 1% was started in addition to natamycin in 17 patients, itraconazole 1% eye ointment was added in addition to natamycin in 18 patients, topical clotrimazole 1% eyedrops (in addition to natamycin alone or natamycin and topical voriconazole) was added in three patients.

With maximal medical therapy, by the final visit, 87 ulcers had a “good” outcome (i.e. 31 healed ulcers and 56 showing improvement by the final visit) and 56 had a “poor” outcome (i.e. 10 perforated, 46 worse or no improvement by the end of the study). Those with a good outcome had slightly shorter symptom duration, smaller ulcer size with fewer deep ulcers at enrolment, compared to those with a poor outcome (Table 2). The main causative organism in those with good outcome was *Fusarium* sp. (n = 43/87) and *Aspergillus* sp. (n = 8/87).

**Table 2:** Baseline characteristics of 143 study participants by clinical outcome.

	Poor Outcome (n = 56)	Good Outcome (n = 87)	p-value^a^
Age, median years (IQR)	50 (37–60)	45 (35–55)	0.148
Gender, no. male, (%)	32 (57%)	62 (71%)	0.082
Symptom duration at presentation, median days, (IQR)	7 (4–15)	6 (4–10)	0.022
Days post-enrolment to final visit, median days (IQR)	16 (10–21)	21 (20–23)	<0.001
Baseline best-corrected visual acuity, logMAR (IQR)	1.8 (0.8–1.8)	1.0 (0.4–1.8)	0.058
Ulcer infiltrate size, median mm, (IQR)^b^	5.0 (3.9–5.9)	3.7 (3.1–4.5)	<0.001
Posterior 1/3 of cornea affected, n (%)	39 (70%)	43 (49%)	0.017

^a^Statistical differences between groups assessed using either chi-squared test for proportions or kruskall wallis test for non-parametrically distributed continuous variables.

^b^Maximum infiltrate size at presentation, prior to corneal scraping.

Of the 56 participants in the “poor” outcome group, 36 had an enlarged stromal infiltrate by the final visit, 10 had no change in the stromal infiltrate from the size at presentation, 10 had developed corneal perforation (of whom two had corneal glue), and a total of 16 patients underwent therapeutic penetrating keratoplasty. The commonest organisms cultured in poor outcome ulcers were *Aspergillus* sp. (n = 16/56, including 12 *Aspergillus flavus*), *Fusarium* sp. (n = 14/56), and dematiaceous fungi (n = 5: *Cylindrocarpon* sp. 1, *Curvularia* sp. 1, *Exserohilum* sp. 2, *Lasiodiplodia* sp. 1). The 10 perforated ulcers were caused by *Fusarium* sp. (n = 2), *Aspergillus flavus* (n = 2), dematiaceous fungi (n = 2: *Cylindrocarpon* sp. and *Exserohilum* sp.) or were culture-negative but light microscopy or IVCM-positive for fungi (n = 4).

### Cellular features in baseline and final visit IVCM images associated with good or poor outcome

The proportion of ulcers with presence of each of the cellular morphologies in baseline and final visit IVCM images are shown in Supplementary Table 1. Logistic regression analysis of baseline images alone showed that poor outcome was significantly associated with the presence of a stellate appearance of interconnected cells without visible nuclei (multivariable OR 2.28, 95% CI: 1.03–5.06, p = 0.043; Table 3), as shown in Fig. 2C.

**Table 3:** Odds ratios (OR) from univariable and multivariable logistic regression models for poor outcome (compared to good outcome) and morphological features in IVCM images from (i) baseline only, (ii) final visit only, or (iii) features that change from being absent in baseline images to being present in final visit images. Models were adjusted for age, gender, baseline ulcer size and time from symptom onset to final visit (for models including final visit data).

Morphological Feature	Univariable OR (95% CI)	p-value	Multivariable OR (95% CI)	p-value
**(i) Baseline visit only**				
Stellate cellular processes no nuclei	2.30 (1.04–5.08)	0. 039	2.28 (1.03–5.06)	0.043
Stromal dendritiform cells	2.99 (0.91–9.80)	0.070	2.93 (0.88–9.67)	0.077
**(ii) Final visit only**				
Fungal hyphae	7.25 (3.09–17.02)	<0.001	6.48 (2.50–16.78)	<0.001
Inflammatory cells (honeycomb)	6.42 (2.07–19.89)	<0.001	5.24 (1.44–19.06)	0.012
Scar	0.17 (0.07–0.40)	<0.001	0.20 (0.08–0.54)	0.001
Inflammatory cells (non-specific distribution)	2.50 (0.93–6.76)	0.070	—	—
(iii) Change from baseline to final visit*				
Fungal filaments	4.34 (2.07–9.10)	<0.001	3.61 (1.64–7.95)	0.001
Stromal dendritiform cells	2.06 (0.89–4.76)	0.090	2.88 (1.17–7.11)	0.022
Stellate cellular processes with no visible nuclei	1.61 (0.93–2.77)	0.088	2.09 (1.14–3.82)	0.017
Stromal scar	0.27 (0.13–0.54)	<0.001	0.22 (0.10–0.49)	<0.001
Inflammatory cells in honeycomb distribution	2.14 (1.28–3.89)	0.013	—	—

*Conditional logistic regression analysis performed comparing changes within each patient from baseline to final visit (i.e. not between patients).

Multivariable analysis of IVCM images at the final visit showed poor outcome to be associated with the presence of inflammatory cells in a honeycomb distribution (OR 5.24, 95% CI: 1.44–19.06, p = 0.012) or intact fungal hyphae (OR 6.48, 95% CI 2.50–16.78, p < 0.001; Table 3). Good outcome was associated with presence of stromal scarring (OR 0.20, 95% CI: 0.08–0.54, p = 0.001; Table 3).

### Changes from baseline to final visit associated with good or poor outcome

Multivariable analysis identified three cellular features that were associated with worsening if they were absent at baseline but were present in final visit IVCM images: fungal filaments (OR 3.61, 95% CI: 1.64–7.95, p = 0.001), stromal stellate cellular appearance with no visible nuclei (OR 2.09, 95% CI: 1.14–3.82, p = 0.017) and dendritiform cells in the stroma (OR 2.88, 95% CI: 1.17–7.11, p = 0.017; Table 3). Change in anterior stromal scar tissue over time, i.e. none at baseline but scarring present at final visit, was the only feature associated with good outcome in this analysis (OR 0.22, 95% CI: 0.10–0.49, p < 0.001, Table 3).

### Disappearance of fungal filaments in IVCM images over time and outcome

**Figure 3:**
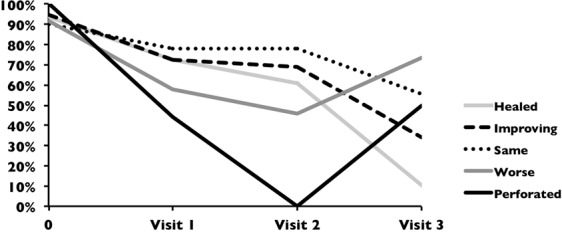
Percentage of fungal keratitis patients with fungal filaments detected in HRT3 *in vivo* confocal microscopy imaging (Y-axis) at baseline and at each follow-up visit after enrolment (i.e. visit 1 at day 7 +/− 3 days; visit 2 at 14 days+/− 3 days or visit 3 at 21 days+/− 3 days, up to 37 days maximum) in the X-axis, for each clinical outcome.

The proportion of patients with fungal filaments detected in IVCM images at each visit categorized by clinical outcome is shown in Fig. 3. In ulcers that healed or had improved by the final visit, there was an initially steep and then consistent decline in the proportion of patients with visible fungal filaments in IVCM images at each sequential visit. However, ulcers that worsened or perforated showed reduction in visible fungal filaments in IVCM images early on after presentation, but then an increase in the proportion that had visible fungi in the IVCM images at the final visit.

Three of the patients whose ulcers had healed by the final visit (n = 29) still had visible fungi in the IVCM images at that time point, as well as evidence of resolving stromal inflammation (i.e. stromal dendritiform cells as well as stellate cellular processes with no visible nuclei in 2, but no inflammatory cells in honeycomb or non-specific distribution visible in any), and normal keratocyte appearances in the stroma in all 3.

### Cellular features associated with corneal perforation

In the univariable regression, no cellular morphologies in baseline or final visit IVCM images were significantly associated with perforation. In terms of change over time (i.e. absence of the cellular feature in baseline IVCM images but presence in the final visit images), two cellular features were associated with perforation in the multivariable analysis: dendritiform cells in the basal epithelial layer appearing in the final visit images (OR 3.31, 95% CI:1.00–10.97, p = 0.05), or a normal keratocyte morphology becoming apparent in the final visit images (OR 4.49, 95% CI: 1.14–17.75, p = 0.032).

### Steroid use

Topical steroid use prior to presentation was not associated with any cellular features in IVCM images in the baseline or final visit. Also, topical steroid use did not have any statistically significant effect on change in any IVCM cellular morphological features between these two visits and was not significantly associated with either good or poor outcome.

## Discussion

Here we have described the cellular changes that occur in the cornea during the clinical course of human FK as observed with the HRT3 IVCM. We found that several cellular or structural morphologies in IVCM images were more likely in ulcers with a poor outcome. The stellate appearance of interconnected cellular processes with absence of visible nuclei, if present in the corneal stroma in baseline images, or development of this appearance in the final visit images, was an indicator of poor outcome. Although we did not conduct histological studies to confirm the cell type associated with this morphological appearance, we compared our images with those from previously published IVCM images and found that this appearance has also been reported post-LASIK^6^, and after collagen-crosslinking^11^. The authors of these studies have postulated that these stellate interconnected cells are most likely activated keratocytes, and the absence of visible nuclei may be due to loss of the nuclei secondary to a cellular event such as apoptosis^6^, or due to water retention causing change in refractive indices between intra-cellular components during corneal oedema, resulting in increased visibility of cellular processes and reduced visibility of the nuclei^12^. Immunohistochemical studies with TUNEL staining have confirmed that keratocyte apoptosis does occur in the human cornea in bacterial, fungal and *Acanthamoeba* keratitis^13,14^. The stellate inter-connected cellular appearance with absent nuclei that we observed in IVCM images, may be a useful warning sign of worsening keratitis - larger studies are required to confirm this finding and histological studies are required to elucidate the cell type corresponding to this morphology.

The presence of an inflammatory cell infiltrate forming a honeycomb distribution in IVCM images at the final visit was also associated with worsening ulcers in our study. A similar morphological appearance has been reported in a mouse model of corneal abrasion injury, where inflammatory cells have been observed to traverse along keratocyte processes in a honeycomb pattern using HRT3 IVCM live imaging (in the anaesthetized mouse), and the inflammatory cell type confirmed as the neutrophil in immunohistochemical examination of the same corneal tissue *ex vivo*^15^. Persistence of neutrophils has been associated with worse clinical outcomes and corneal perforation in BK and FK, most likely due to their ability to release tissue-damaging enzymes (e.g. MMP9) and pro-inflammatory cytokines (e.g. IL1B) that perpetuate the host inflammatory response^16–18^.

Another feature that was associated with worse outcome was the detection of fungal hyphae in final visit IVCM images. Persistence of fungi even at later stages of keratitis may be due to inadequate host mechanisms to clear the infection; one such mechanism may be through production of molecules that are able to subvert the host immune response, e.g. alkaline phosphatase-1 secreted by *Aspergillus* sp. that is able to destroy host complement proteins C3 and C4^19^. Also, poor penetration of natamycin eye drops into the deeper cornea, and poor efficacy of this medication against some fungal species such as *Aspergillus* sp., may contribute to persistence of fungi in the deep cornea in late-stage keratitis^20,21^.

We found that corneal perforation was associated with the appearance of dendritiform cells in the basal epithelial layer in the final visit, or also the appearance of normal keratocyte morphology in final visit image. This implies that the fungi in these severe ulcers are able to grow through the cornea successfully with only a superficial immune response triggered in the dendritiform cells in the epithelium, and without triggering any response in the local keratocytes. Histological studies of corneal tissue excised at the time of corneal transplantation surgery in severe FK have shown that regions of the tissue with a high fungal load frequently do not have any inflammatory cell infiltration^22^. Possible reasons for this may be lack of penetration of natamycin into the deeper cornea, or the fungal subversion of the host response as outlined above. Corneal perforation may then occur in these cases due to fungal proteases damaging corneal collagens, including those in Descemet’s membrane^23,24^.

The appearance of dendritiform cells in the stroma at the final visit, when not present at the baseline visit, was also associated with ulcer worsening. These cells may represent dendritic cells or macrophages that have been recruited to the ulcer towards the later stages of disease, or may also represent fibroblasts that are involved in wound repair at the site of the ulcer^25–27^. Further comparative histological studies are required to identify the cell type forming this morphological appearance in the IVCM images, in order to understand the role these cells may play in the worsening of disease.

One limitation of our study is that although we have identified IVCM cellular appearances, there are potentially multiple cell types that can form these morphologies. For example, cells with a dendritiform appearance in IVCM images may be macrophages or dendritic cells that reside within the cornea, but may also be bone-marrow derived myeloid cells that have migrated into the inflamed tissue^27,28^. Correlated images using both IVCM and immunohistochemistry within the same tissue sample are required in future studies in order to identify cell types and their morphology. Our study has focused on large ulcers and so findings are mainly applicable to moderate-to-severe keratitis; further research is required in ulcers at an earlier stages of presentation in order to identify whether the same cellular morphologies that we have found are also present in early disease.

Another limitation is that the HRT3 IVCM allows high resolution imaging of the living cornea, but with a limited field of view, and so requires multiple images to be taken of the ulcer margins to build up an overall picture of cellular changes in the whole ulcer^1^. As such an experienced operator is required so that the best set of images possible are captured. Also, an experienced observer is required to successfully identify the morphological features that we have described in our study, and so training would be required. We did not assess inter- or intra-grader agreement in this study, as only a single observer was used, however our prior report has shown there is variability in inter-grader agreement based upon grader experience^2^. Hence further studies are required to not only validate IVCM image-based prognostic markers, but to also assess the diagnostic accuracy of graders in detecting these morphologies. A major limitation of IVCM at present is that the current techniques do not allow exact re-imaging of the same cellular location in the cornea at future follow-up visits. In this study we have attempted to image as much of the ulcer as possible with multiple volume scans, to try to image as much of the ulcer as possible from epithelium to endothelium. Experimental methods are currently underway to combine anterior segment OCT techniques with HRT3 IVCM to attempt to be able to co-locate the imaging location within the cornea, and so future combined imaging modalities such as this may help to be able to serially assess pathological changes in the cornea over time^29^.

The alternative IVCM system available, Confoscan, provides a motorized lens that allows rapid imaging of the entire corneal ulcer quickly for less experienced operators, but will provide lower resolution images, with less detail of the keratocyte intracellular changes. However, use of the white light-based Confoscan system (Nidek technologies, Japan) in FK in high prevalence settings has in some studies allowed identification of resolution of inflammatory cell infiltrates and fungal filaments^5^, and so further research is required to assess morphological parameters in images from this system and whether they may correlate with clinical outcome. Future studies could also consider automation of the assessment of cellular features, such as cell counting to estimate keratocyte cell density, or size of dendritiform cells, or to assess the fungal filament load in each images. Automation using computational analysis would make such assessments much easier for the inexperienced observer, and also in the research setting since the number of total images from all volume scans are often too large to allow for manual cell counting or fungal filament tracing (>65,000 images screened in this study alone).

In summary, we have found that several cellular morphologies as detected with HRT3 IVCM may be associated with worsening versus healing ulcers. Since it can often be difficult to monitor response to treatment with clinical examination alone, these specific IVCM features should be further investigated as possible prognostic biomarkers.

## Supplementary information

Supplementary Table 1

## Data Availability

The datasets generated and/or analysed during the current study are available from the corresponding author on reasonable request.
