# Supplementary material for: Cellular morphological changes detected by laser scanning in vivo confocal microscopy associated with clinical outcome in fungal keratitis
Source: Sci Rep. 2019 Jun 6;9:8334. doi: 10.1038/s41598-019-44833-9 (PMC6554396; doi:10.1038/s41598-019-44833-9)
Supplement: Supplementary file 1 — Supplementary Table 1 [file 41598_2019_44833_MOESM1_ESM.pdf]

Cellular morphological changes detected by laser scanning *in vivo* confocal microscopy associated with clinical outcome in fungal keratitis

Jaya D Chidambaram, MBBS, MRCOphth, PhD[1*], Namperumalsamy V Prajna, DNB, FRCOphth[2,3], Srikanthi Palepu, MD[2], Shruti Lanjewar, MD[2], Manisha Shah, MD[2,3], Shanmugam Elakkiya, MSc[3], Prajna Lalitha, MD[2,3], David Macleod, PhD[1], Matthew J. Burton, FRCOphth, PhD.[1,4]

[1]London School of Hygiene & Tropical Medicine, Keppel Street, London WC1E 7HT, UK

[2]Aravind Eye Hospital, 1 Anna Nagar, Madurai 625 020, Tamil Nadu, India

[3]Aravind Medical Research Foundation, Kuruvikaran Salai, Anna Nagar, Shenoy Nagar, Madurai 625020, Tamil Nadu, India

[4]Cornea Department, Moorfields Eye Hospital, 162 City Road, London EC1V 2PD, UK

[*]Corresponding Author: Dr. Jaya D. Chidambaram, International Centre for Eye Health, London School of Hygiene & Tropical Medicine, Keppel Street, London WC1E 7HT, UK. Tel: +44-(0)207-958-8343. Email: Jaya.Chidambaram@Lshtm.ac.uk.

Supplementary Table 1: Corneal morphological features detected at the baseline and final visits in IVCM images.

IVCM Feature	Baseline		Final Visit	
	**Poor Outcome (n=56)**	**Good Outcome (n=87)**	**Poor Outcome (n=56)**	**Good Outcome (n=87)**
Epithelial bullae	6 (11%)	7 (8%)	5 (9%)	7 (8%)
Stromal bullae	4 (7%)	9 (10%)	23 (41%)	53 (61%)
Basal DCs	29 (52%)	46 (53%)	33 (59%)	56 (64%)
Stromal DCs	9 (16%)	7 (8%)	7 (12%)	7 (8%)
Inflammatory cells (Honeycomb)	29 (52%)	45 (52%)	20 (36%)	5 (6%)
Inflammatory cells (Non-specific)	16 (29%)	25 (29%)	15 (27%)	9 (10%)
Normal keratocytes	41 (73%)	70 (80%)	17 (30%)	27 (31%)
Stellate cellular processes with nuclei	33 (59%)	65 (75%)	34 (61%)	61 (70%)
Granules	35 (62%)	47 (54%)	14 (25%)	22 (25%)
Stellate cellular processes no nuclei	24 (43%)	23 (26%)	21 (37%)	31 (36%)
Spindles	39 (70%)	65 (75%)	34 (61%)	60 (69%)
Scar	5 (9%)	10 (11%)	16 (29%)	64 (74%)
Intact fungal filaments	52 (93%)	82 (94%)	41 (73%)	23 (26%)
Broken fungal filaments	13 (23%)	16 (18%)	10 (18%)	5 (6%)
